# The association of chronic liver disorders with exacerbation of symptoms and complications related to COVID‐19: A systematic review and meta‐analysis of cohort studies

**DOI:** 10.1111/crj.13552

**Published:** 2022-10-18

**Authors:** Maryam Afraie, Pardis Mohammadzedeh, Mobin Azami, Sorour Khateri, Kamran Zamani, Farhad Moradpour, Yousef Moradi

**Affiliations:** ^1^ Department of Epidemiology and Biostatistics, Faculty of Medicine Kurdistan University of Medical Sciences Sanandaj Iran; ^2^ Student Research Committee Kurdistan University of Medical Sciences Sanandaj Iran; ^3^ Department of Physical Medicine and Rehabilitation Hamedan University of Medical Sciences Hamedan Iran; ^4^ Social Determinants of Health Research Center, Research Institute for Health Development Kurdistan University of Medical Sciences Sanandaj Iran

**Keywords:** chronic liver disease, complications, COVID‐19, meta‐analysis, mortality

## Abstract

**Introduction:**

The aim of this review was to combine the results of published cohort studies to determine the exact association between chronic liver disorders, and the severe form of COVID‐19, and its associated complications.

**Methods:**

This meta‐analysis employed a keyword search (COVID‐19 and chronic liver disorders) using PubMed (Medline), Scopus, Web of Sciences, and Embase (Elsevier). All articles related from January 2019 to May 2022 were reviewed. The STATA software was used for analysis.

**Results:**

The risk of death in COVID‐19 patients with chronic liver disorders was higher than in ones without the chronic liver disease (RR: 1.52; CI 95%: 1.46–1.57; *I* ^2^: 86.14%). Also, the risk of acute respiratory distress syndrome (ARDS) and hospitalization in COVID‐19 patients with chronic liver disorders was higher than in ones without the chronic liver disease ([RR: 1.65; CI 95%: 1.09–2.50; *I* ^2^: 0.00%] and [RR: 1.39; CI 95%: 1.23–1.58; *I* ^2^: 0.20%]). Also, the meta‐analysis showed cough, headache, myalgia, nausea, diarrhea, and fatigue were 1.37 (CI 95%: 1.20–1.55), 1.23 (CI 95%: 1.09–1.38), 1.25 (CI 95%: 1.04–1.50), 1.19 (CI 95%: 1.02–1.40), 1.89 (CI 95%: 1.30–2.75), 1.49 (CI 95%: 1.07–2.09), and 1.14 (CI 95%: 0.98–1.33), respectively, whereas the risk of all these symptoms was higher in COVID‐19 patients with chronic liver diseases than ones without chronic liver disorders.

**Conclusion:**

The mortality and complications due to COVID‐19 were significantly different between patients with the chronic liver disease and the general population.

## Graphical abstract

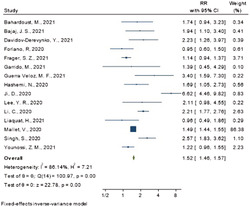

## 1 INTRODUCTION

The chronic liver disease (CLD) is a progressive deterioration of liver function over 6 months, which slowly progresses, leaving the liver unable to synthesize coagulation factors, and proteins, detoxify harmful metabolic products, and excrete bile.^1^, ^2^ It is a continuous process of inflammation, destruction, and regeneration of the liver parenchyma, which leads to fibrosis and cirrhosis.^1^, ^3^ CLD is caused by a wide range of causes, including toxins, long‐term alcohol abuse, infection, autoimmune diseases, genetic disorders, and metabolic disorders.^1^, ^4^ Cirrhosis is the final stage of CLD, which leads to liver dysfunction, extensive nodule formation, vascular reorganization, neo‐angiogenesis, and extracellular matrix deposition.^1^, ^5^, ^6^ The underlying mechanism of fibrosis and cirrhosis at the cellular level is the uptake of stellate cells and fibroblasts, which leads to fibrosis while parenchymal regeneration relies on liver stem cells.^1^, ^7^ The CLD is a very common clinical condition and the focus is more on its common causes, clinical manifestations and management.^1^, ^8^, ^9^, ^10^, ^11^ About 1.5 billion people worldwide have CLD, which causes more than 2 million deaths a year.^9^, ^12^ With the rapid spread of COVID‐19, there are considerable concerns that patients with CLD are a vulnerable population and at higher risks for the more severe form of COVID‐19 and its associated complications.^13^ Early on, patients with underlying diseases such as diabetes, chronic lung diseases, cardiovascular diseases, hypertension, and cancer were labeled as those at high risks for severe COVID‐19.^14^ Although the virus mainly affects the lungs, experiences of China and the United States suggest SARS‐CoV‐2 may affect extra‐pulmonary systems, including the gastrointestinal and hepato‐biliary systems.^15^, ^16^

However, it was not initially clear whether patients with the CLD were more susceptible to COVID‐19. Data from some recent studies showed the CLD in the absence of immunosuppressive therapy was not associated with an increased risk of COVID‐19,^17^ whereas results from other studies showed the CLD, including patients with cirrhosis and non‐cirrhosis, was associated with higher rates of mortality because of COVID‐19.^18^, ^19^, ^20^ Also, various reports from different countries showed more than half of the hospitalized adults due to COVID‐19 had abnormal aminotransferase levels and 2% to 11% of them had underlying liver diseases.^17^, ^21^, ^22^, ^23^, ^24^, ^25^ However, there are limited and conflicting reports on the liver disease nature among COVID‐19 patients, and it is unclear how underlying CLD affects liver injuries and clinical outcomes in these patients.^26^ Due to the discrepancies in the results of previous studies and the importance of determining the association between various underlying diseases, especially liver disorders, and COVID‐19 and its severe form, the authors in this meta‐analysis decided to combine the results of published cohort studies to determine the exact association between chronic liver disorders, and the severe form of COVID‐19, and its associated complications.

## 2 METHODS

The guideline of Preferred Reporting Items for Systematic Reviews and Meta‐Analyses (PRISMA) was used to review meta‐analyses, and systematic reviews.^27^ Also, the study protocol was registered in PROSPER with the code CRD42022327806.

### 2.1 Search strategy and screening

The search was performed without language restrictions. The search strategy consisted of the following main keywords extracted from Mesh:

“COVID 19,” “Liver Disease,” and “Chronic Liver Dysfunction.”

Search databases included PubMed (Medline), Scopus, Web of Sciences, and Embase (Elsevier).

The search deadline was from January 1, 2019, to January 1, 2022. Duplicate published articles were removed considering their titles, authors, and publication years using Endnote software version 9. Then, the remaining studies were evaluated by reviewing their titles, abstracts, and full texts, considering the inclusion criteria. In addition to searching the mentioned databases, gray literature was searched by reviewing articles in the first 10 pages of Google scholar, and manual search was performed by reviewing references of related studies. Two authors (MA, MA) independently screened articles based on their titles, abstracts, and full texts, and disputes were resolved by the third one (YM). After screening, the studies were finally selected by evaluating their full texts.

### 2.2 Inclusion and exclusion criteria

The inclusion criteria were defined based on the PECOT structure as follows:

Population included the whole population, whether people with the CLD or healthy ones, exposure was considered as the presence of chronic liver disorders, comparison included comparing healthy people with those with chronic liver disorders, and outcomes included the COVID‐19 symptoms and its associated complications, such as hospitalization in the intensive care unit, death, and acute respiratory distress syndrome (ARDS). The intended studies to conduct meta‐analysis were cohort ones because this type of observational studies is much more important than others to examine the casual association.

Systematic reviews, case reports, case series, case controls, cross sectionals, clinical trials, and other interventional studies as well as letters to the editor were excluded from the present research. Also, studies that met the inclusion criteria but their full texts were not available, first by sending an email to their authors, the full texts were requested and if the authors did not respond, they were removed.

### 2.3 Data extraction

After three stages of evaluation of titles, abstracts, and full texts, the selected articles were retrieved for detailed analysis. Data were collected using a checklist included authors' names, country, publication year, study type, study population, sample size, data source, age, number of patients with COVID‐19, hospitalization and mortality due to COVID‐19, ARDS, need for ventilation, ICU hospitalization, and evaluation of COVID‐19 symptoms (fever, cough, weakness, chest pain, abdominal pain, CT scan, Chest X‐ray, respiratory problems and shortness of breath, decreased sense of smell decreased sense of taste, fatigue, headache, dizziness, myalgia, diarrhea, nausea, and vomiting).

### 2.4 Risk of bias

Two of the authors (MA and PM) conducted a qualitative evaluation of the studies based on the Newcastle–Ottawa Quality Assessment Scale (NOS) checklist designed to evaluate the quality of observational studies. This tool examines each research with eight items in three groups, including how to select study samples, how to compare and analyze study groups, and how to measure and analyze the desired outcome. Each of these items is given a score of one if it is observed in the studies, and the maximum score for each study is 9 points. In case of discrepancies in the score assigned to the published articles, the discussion method and the third researcher were applied to reach an agreement.

### 2.5 Statistical analysis

To calculate the association, cumulative risk ratio (RR) with the 95% confidence interval and the meta set command were used considering logarithm and logarithm standard deviation of the RR. Heterogeneity was assessed between studies using the *I* ^2^ and Q Cochrane tests. Egger test was used to evaluate the publication bias. Statistical analysis was performed using STATA 16.0 and *P*‐value < 0.05 was considered.

## 3 RESULTS

**FIGURE 1:**
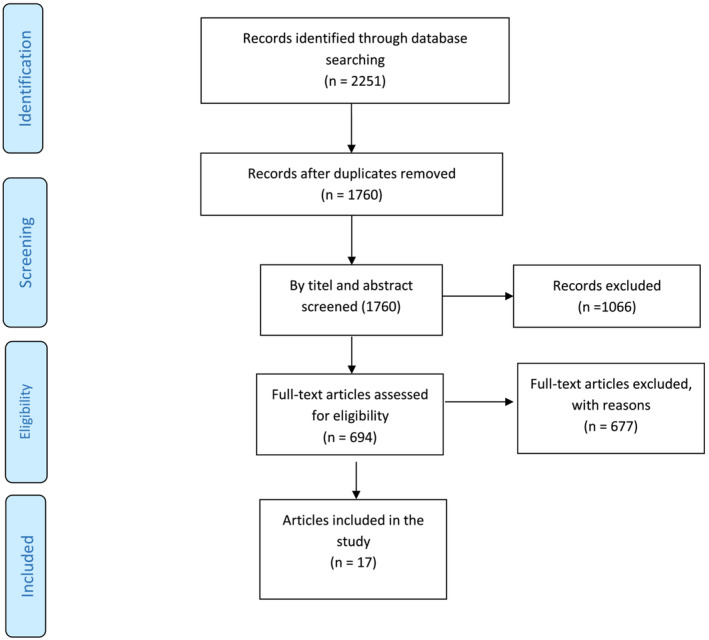
The flowchart of search strategy and syntax

First, 2251 studies were collected by searching based on the search strategy in the desired databases, of which 491 studies were duplicated, and 1760 ones remained. After reviewing the remained articles based on their titles, abstracts and full texts, 17 studies were selected for analysis. All articles were conducted in 2020 and 2021 and were cohorts (Figure 1 and Tables 1 and 2).

**TABLE 1:** Characteristics of included cohort studies (items related to COVID‐19 complications)

Ref.	Authors (years) country	Type of study	Study population	Sample size	Data sources	Age (male)	Number of COVID‐19	Admission to hospital (COVID‐19)	Mortality (COVID‐19)	ARDS	ICU	Ventilation
^28^	Bahardoust, M. (2021) (Iran)	Cohort	Liver disease + Covid‐19 Covid‐19	Liver disease = 81 Non‐liver disease = 921	Hospital	62.2	Liver disease = 81 Non‐liver disease = 921	Liver disease = 81 Non‐liver disease = 921 Liver disease = 76 non‐liver disease = 598 >7 day	Liver disease = 10 non‐liver disease = 65	NR	NR	NR
^29^	Bajaj, J. S. (2021) (use)	Cohort	Covid‐19 alone Covid‐19 + Cirrhosis Cirrhosis alone	COVID‐19 alone = 108 COV + Cirrhosis = 37 Cirrhosis alone = 127	Hospital	61.3	COVID‐19 alone = 108 COVID‐19 + cirrhosis = 37	COVID‐19 alone = 108 COVID‐19 + cirrhosis = 37 Cirrhosis alone = 127	Covid‐19 = 15 Covid‐19 + Cirrhosis = 11 Cirrhosis = 24	NR	Covid‐19 = 41 Covid‐19 + Cirrhosis = 16 Cirrhosis = 31	Covid‐19 = 41 COVID‐19 + Cirrhosis = 14 Cirrhosis = 18
^30^	Davidov‐Derevynko, Y. (2021) (Israel)	Cohort	COVID + Liver COVID + non‐Liver	Covid‐19 = 323 Covid‐19 + Liver = 59	hospital	59.1	COVID‐19 = 323 COVID‐19 + Liver = 59	COVID‐19 = 323 COVID‐19 + Liver = 59	COVID‐19 = 22 COVID‐19 + Liver = 10	NR	COVID‐19 = 36 COVID‐19 + Liver = 9	COVID‐19 = 35 COVID‐19 + Liver = 9
^31^	Forlano, R. (2020) (England)	Cohort	NAFLD + Covid‐19 Non‐NAFLD + Covid‐19	NAFLD + Covid‐19 = 61 Covid‐19 = 132	Hospital	70.5	NAFLD +Covid‐19 = 61 Covid‐19 = 132	NAFLD +Covid‐19 = 61 Covid‐19 = 132	NAFLD + Covid‐19 = 18 Covid‐19 = 41	NR	NAFLD + Covid‐19 = 11 Covid‐19 = 27	NR
^32^	Frager, S. Z. (2021) (USA)	Cohort	Covid‐19 id Covid‐19 + Liver	Covid‐19 = 2895 Covid‐19 + Liver = 457	Hospital	64.8	Covid‐19 = 2895 Covid‐19 + Liver = 457	Covid‐19 = 2895 Covid‐19 + Liver = 457	Covid‐19 = 769 Covid‐19 + Liver = 135	NR	NR	Covid‐19 = 522 Covid‐19 + Liver = 108
^33^	Garrido, M. (2021) (Portugal)	Cohort	Covid‐19 id Covid‐19 + Liver	Covid‐19 = 303 Covid‐19 + Liver = 14	Hospital	70.5	Covid‐19 = 303 Covid‐19 + Liver = 14	Covid‐19 = 303 Covid‐19 + Liver = 14	Covid‐19 = 67 Covid‐19 + Liver = 4	NR	NR	Covid‐19 = 18 Covid‐19 + Liver = 1
^34^	Guerra Veloz, M. F.(2021) (Spain)	cohort	Covid‐19 Covid‐19 + Liver	Covid‐19 = 419 Covid‐19 + Liver = 28		40.8	Covid‐19 = 419 Covid‐19 + Liver = 28	Covid‐19 = 200 Covid‐19 + Liver = 26	Covid‐19 = 39 Covid‐19 + Liver = 8		Covid‐19 = 28 Covid‐19 + Liver = 3	Covid‐19 = 19 Covid‐19 + Liver = 2
^26^	Hashemi, N. (2020) (USA)	Cohort	Covid‐19 Covid‐19 + Liver	Covid‐19 = 294 Covid‐19 + Liver = 69	Hospital	64.8	Covid‐19 = 294 Covid‐19 + Liver = 69	Covid‐19 = 294 Covid‐19 + Liver = 69	Covid‐19 = 39 Covid‐19 + Liver = 16	NR	Covid‐19 = 103 Covid‐19 + Liver = 34	Covid‐19 = 89 Covid‐19 + Liver = 33
^35^	Huang, R. (2020) (China)	Cohort	Non‐NAFLD + Covid‐19 NAFLD + Covid‐19	Covid‐19 = 194 Covid‐19 + Liver = 86	Hospital	43.5	Covid‐19 = 194 Covid‐19 + Liver = 86	Covid‐19 = 194 Covid‐19 + Liver = 86	Covid‐19 = 0 Covid‐19 + Liver = 0	Covid‐19 = 2 Covid‐19 + Liver = 2	Covid‐19 = 13 Covid‐19 + Liver = 5	NR
^36^	Ji, D. (2020) (China)	Cohort	Covid‐19 Covid‐19 + Liver	Covid‐19 = 118 Covid‐19 + Liver = 22	Hospital	63.6	Covid‐19 = 118 Covid‐19 + Liver = 22	Covid‐19 = 118 Covid‐19 + Liver = 22	Covid‐19 = 0 Covid‐19 + Liver = 1	NR	NR	NR
^37^	Lee, Y. R. (2020) (South Korea)	Cohort	Covid‐19 Covid‐19 + Liver	COVID‐19 + Liver = 47 COVID‐19 = 958	Hospital	55.3	COVID‐19 + Liver = 47 COVID‐19 = 958	COVID‐19 + Liver = 47 COVID‐19 = 958	COVID‐19 + Liver = 7 COVID‐19 = 70	COVID‐19 + Liver = 8 COVID‐19 = 105	COVID‐19 + Liver = 8 COVID‐19 = 89	Invasive mechanical ventilation COVID‐19 + Liver = 4 COVID‐19 = 66 Invasive mechanical ventilation and ECMO COVID‐19 + Liver = 1 COVID‐19 = 17
^38^	Li, C. (2020) (China)	Cohort	Covid‐19 Covid‐19 + Liver	COVID‐19 + Liver = 52 Covid‐19 = 52	Hospital	58.2	COVID‐19 + Liver = 52 COVID‐19 = 52	COVID‐19 + Liver = 52 COVID‐19 = 52	COVID‐19 + Liver = 9 COVID‐19 = 0	NR	NR	Non‐invasive ventilation COVID‐19 = 2 COVID‐19 + Liver = 5 Invasive mechanical ventilation COVID‐19 = 1 COVID‐19 + Liver = 5 NO ventilation COVID‐19 = 49 COVID‐19 + Liver = 42
^39^	Liaquat, H. (2021) (USA)	Cohort	COVID‐19 COVID‐19 + Liver	COVID‐19 = 105 COVID‐19 + Liver = 34	Hospital	73.9	COVID‐19 = 105 COVID‐19 + Liver = 34	COVID‐19 = 105 COVID‐19 + Liver = 34	COVID‐19 = 29 COVID‐19 + Liver = 9	COVID‐19 = 41 COVID‐19 + Liver = 19	COVID‐19 = 25 COVID‐19 + Liver = 14	COVID‐19 = 11 COVID‐19 + Liver = 7
^40^	Mallet, V. (2020) (France)	Cohort	COVID‐19 COVID‐19 + Liver	COVID‐19 = 243 634 COVID‐19 + Liver = 15 476	Hospital	70	COVID‐19 = 243 634 COVID‐19 + Liver = 15 476	COVID‐19 = 243 634 COVID‐19 + Liver = 15 476	COVID‐19 = 35 262 COVID‐19 + Liver = 2941	NR	NR	COVID‐19 = 16 449 COVID‐19 + Liver = 1600
^41^	Ge, J. (2020) (USA)	Cohort	1. Noncirrhosis/negative 2. Non‐cirrhosis/positive; 3. Cirrhosis/negative; 4. Cirrhosis/positive	1. Healthy = 128 864 2. COVID‐19 = 29 446 3. Cirrhosis = 53 476 4. Cirrhosis + Covid‐19 = 8941	Pop	53	2. COVID‐19 = 29 446 4. Cirrhosis + Covid‐19 = 8941	NR	NR	NR	NR	NR
^19^	Singh, S. (2020) (USA)	Cohort	Covid‐19 + Liver Covid‐19	All = 2780 Covid‐19 + Liver = 250 Covid‐19 = 2530	Pop	55.2	All = 2780 Covid‐19 + Liver = 250 Covid‐19 = 2530	Covid‐19 + Liver = 130 Covid‐19 = 760	Covid‐19 + Liver = 30 Covid‐19 = 110	NR	NR	NR
^42^	Younossi, Z. M. (2021) (USA)	Cohort	NAFLD + Covid‐19 Non‐NAFLD + Covid‐19	NAFLD = 553 Non‐NAFLD = 2736	Hospital	54.7	NAFLD = 553 Non‐NAFLD = 2736	NAFLD = 553 Non‐NAFLD = 2736	NAFLD = 60 Non‐NAFLD = 239	NR	NAFLD = 196 Non‐NAFLD = 726	NAFLD = 76 Non‐NAFLD = 221

**TABLE 2:** Characteristics of included cohort studies (items related to signs and symptoms of COVID‐19)

	Fever	Cough	Chest pain	Dizziness	Headache	Myalgia	Diarrhea	Vomiting	Test sense	Shortness of breath	Abdominal pain	Fatigue	Dyspnea
^28^	Liver disease ≥38.5 = 35 <38.5 = 46 Non‐liver disease ≥38.5 = 175 <38.5 = 746	Liver disease = 47 Non‐liver disease = 552	Liver disease = 20 Non‐liver disease = 221	Liver disease = 20 Non‐liver disease = 313	Liver disease = 26 Non‐liver disease = 275	Liver disease = 49 Non‐liver disease = 485	Liver disease = 39 Non‐liver disease = 230	Liver disease = 37 Non‐liver disease = 276	Liver disease = 13 Non‐liver disease = 166	NR	NR	NR	NR
^29^	Covid‐19 = 75 Covid‐19 + Cirrhosis = 20	Covid‐19 = 70 Covid‐19 + Cirrhosis = 26	Covid‐19 = 19 Covid‐19 + Cirrhosis = 3	Covid‐19 = 8 Covid‐19 + Cirrhosis = 4	Covid‐19 = 15 Covid‐19 + Cirrhosis = 2	Covid‐19 = 31 Covid‐19 + Cirrhosis = 4	Covid‐19 = 29 Covid‐19 + Cirrhosis = 3	Covid‐19 = 29 Covid‐19 + Cirrhosis = 4	NR	Covid‐19 = 74 Covid‐19 + Cirrhosis = 23	Covid‐19 = 17 Covid‐19 + Cirrhosis = 9	Covid‐19 = 44 Covid‐19 + Cirrhosis = 12	NR
^33^	Covid‐19 = 174 Covid‐19 + Liver = 8	Covid‐19 = 185 Covid‐19 + Liver = 10	NR	NR	Covid‐19 = 46 Covid‐19 + Liver = 0	Covid‐19 = 69 Covid‐19 + Liver = 2	Covid‐19 = 38 Covid‐19 + Liver = 2	Covid‐19 = 23 Covid‐19 + Liver = 1	NR	NR	Covid‐19 = 17 Covid‐19 + Liver = 2	Covid‐19 = 82 Covid‐19 + Liver = 3	Covid‐19 = 108 Covid‐19 + Liver = 6
^35^	Covid‐19 = 132 Covid‐19 + Liver = 55	Covid‐19 = 109 Covid‐19 + Liver = 47	NR	NR	Covid‐19 = 16 Covid‐19 + Liver = 3	Covid‐19 = 21 Covid‐19 + Liver = 7	NR	NR	NR	Covid‐19 = 16 Covid‐19 + Liver = 7	NR	Covid‐19 = 42 Covid‐19 + Liver = 16	NR
^37^	COVID‐19 + Liver = 24 COVID‐19 = 425	COVID‐19 + Liver = 23 COVID‐19 = 544	NR	NR	COVID‐19 + Liver = 8 COVID‐19 = 245	COVID‐19 + Liver = 9 COVID‐19 = 316	NR	NR	NR	COVID‐19 + Liver = 15 COVID‐19 = 232	NR	NR	NR
^42^	NAFLD = 319 Non‐NAFLD = 1283	NAFLD = 318 Non‐NAFLD = 1273		NAFLD = 23 Non‐NAFLD = 72	NAFLD = 74 Non‐NAFLD = 240	NAFLD = 101 Non‐NAFLD = 363	NR	NR	NAFLD = 23 Non‐NAFLD = 72	NAFLD = 316 Non‐NAFLD = 1519		NAFLD = 144 Non‐NAFLD = 588	

**FIGURE 2:**
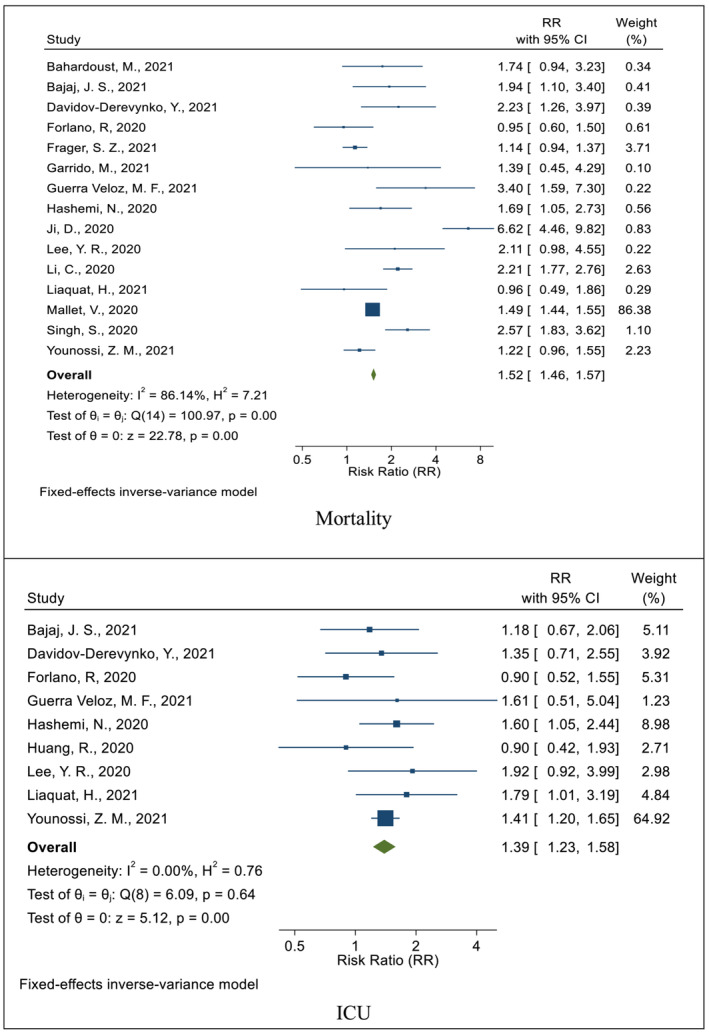
The pooled effect (risk ratio, RR) of chronic liver disorders on the mortality and admission to ICU related to COVID‐19

The risk of death in COVID‐19 patients with chronic liver disorders was compared with that in ones without the CLD in 15 studies.^19^, ^26^, ^28^, ^29^, ^30^, ^31^, ^32^, ^33^, ^34^, ^35^, ^36^, ^37^, ^38^, ^39^, ^40^, ^42^ In all of these studies, the mortality rate in patients with COVID‐19, and the CLD was higher than that in patients with COVID‐19, and without the CLD.^19^, ^26^, ^28^, ^30^, ^32^, ^33^, ^34^, ^36^, ^37^, ^38^, ^39^, ^40^ The cumulative RR after combining these studies was 1.52 (CI 95%: 1.46–1.57; *I* ^2^: 86.14%) (Figure 2).

The risk of ARDS in patients with COVID‐19 who had chronic liver disorders was compared in three studies with those without chronic liver disorders.^35^, ^37^, ^39^ The cumulative RR after combining these studies was 1.65 (CI 95%: 1.09–2.50; *I* ^2^: 0.00%) (Table 3).

**TABLE 3:** Results of meta‐analysis and reports pooled odds ratio based on symptoms and complications related to COVID‐19

	Outcomes	No. of study (SS)	Sample size liver disease	Sample size non‐liver disease	No. of Covid‐19				Pooled RR	Heterogeneity assessment		
					A	B	C	D		*I* ^2^	*P*‐value	Test Q
Outcomes of Covid‐19	Mortality	15 (272767)	17 240	255 527	3269	33 767	13 971	221 760	1.52 (1.46–1.57)	86.14%	0.00	100.97
	ARDS	3 (1424)	167	1257	29	148	138	1109	1.65 (1.09–2.50)	0.00%	1.00	0.00
	ICU	9 (6243)	974	5269	296	1088	678	4181	1.39 (1.23–1.58)	0.00%	0.31	0.64
	Ventilation	11 (268653)	16 826	251 827	1864	17 474	14 962	234 353	1.53 (1.46–1.60)	0.00%	0.86	5.50
Covid‐19 symptoms	Fever	6 (6037)	818	5219	461	2264	357	2955	1.37 (1.20–1.55)	81.32%	0.00	26.77
	Cough	8 (6280)	904	5376	536	2849	368	2527	1.23 (1.09–1.38)	46.65%	0.07	13.12
	Chest pain	2 (1146)	118	1028	23	240	95	788	0.92 (0.59–1.42)	32.41%	0.22	1.48
	Dizziness	3 (4435)	671	3764	47	393	624	3371	1.12 (0.85–1.48)	70.49%	0.03	6.78
	Headache	5 (6037)	818	5219	113	837	705	4382	1.25 (1.04–1.50)	64.72%	0.02	11.34
	Myalgia	6 (6037)	818	5219	172	1285	646	3934	1.19 (1.02–1.40)	67.92%	0.01	15.59
	Diarrhea	3 (1463)	132	1331	44	297	88	1034	1.89 (1.30–2.75)	83.88%	0.00	12.41
	Vomiting	3 (1463)	132	1331	42	328	90	1003	1.44 (0.99–2.09)	75.22%	0.02	8.07
	Test sense	2 (4290)	634	3656	36	238	598	3418	1.26 (0.93–1.71)	53.75%	0.14	2.16
	Shortness of breath	4 (4719)	723	3996	361	1841	362	2155	1.05 (0.92–1.21)	0.00%	0.60	1.89
	Abdominal pain	2 (462)	51	411	11	34	40	377	1.61 (0.91–2.85)	0.00%	0.47	0.53
	Fatigue	4 (4031)	690	3341	175	756	515	2585	1.14 (0.98–1.33)	28.31%	0.24	4.18
	Dyspnea	3 (560)	100	460	49	198	51	262	1.49 (1.07–2.09)	0.00%	0.96	0.08

*Note*: A: COVID‐19 individual with CLD; B: Healthy individual with CLD; C: COVID‐19 individual without CLD; D: Healthy individual without CLD.

The risk of hospitalization of COVID‐19 patients who had chronic liver disorders in the intensive care unit was compared with that of ones without the CLD in nine studies,^26^, ^29^, ^30^, ^31^, ^34^, ^35^, ^37^, ^39^, ^42^, ^43^ in all of which the risk of hospitalization in the ICU was higher in the group of COVID‐19 patients with the CLD.^26^, ^29^, ^30^, ^31^, ^34^, ^35^, ^37^, ^39^ The cumulative RR after combining these studies was 1.39 (CI 95%: 1.23–1.58) with heterogeneity (*I* ^2^) of 0.2% (Figure 2).

**FIGURE 3:**
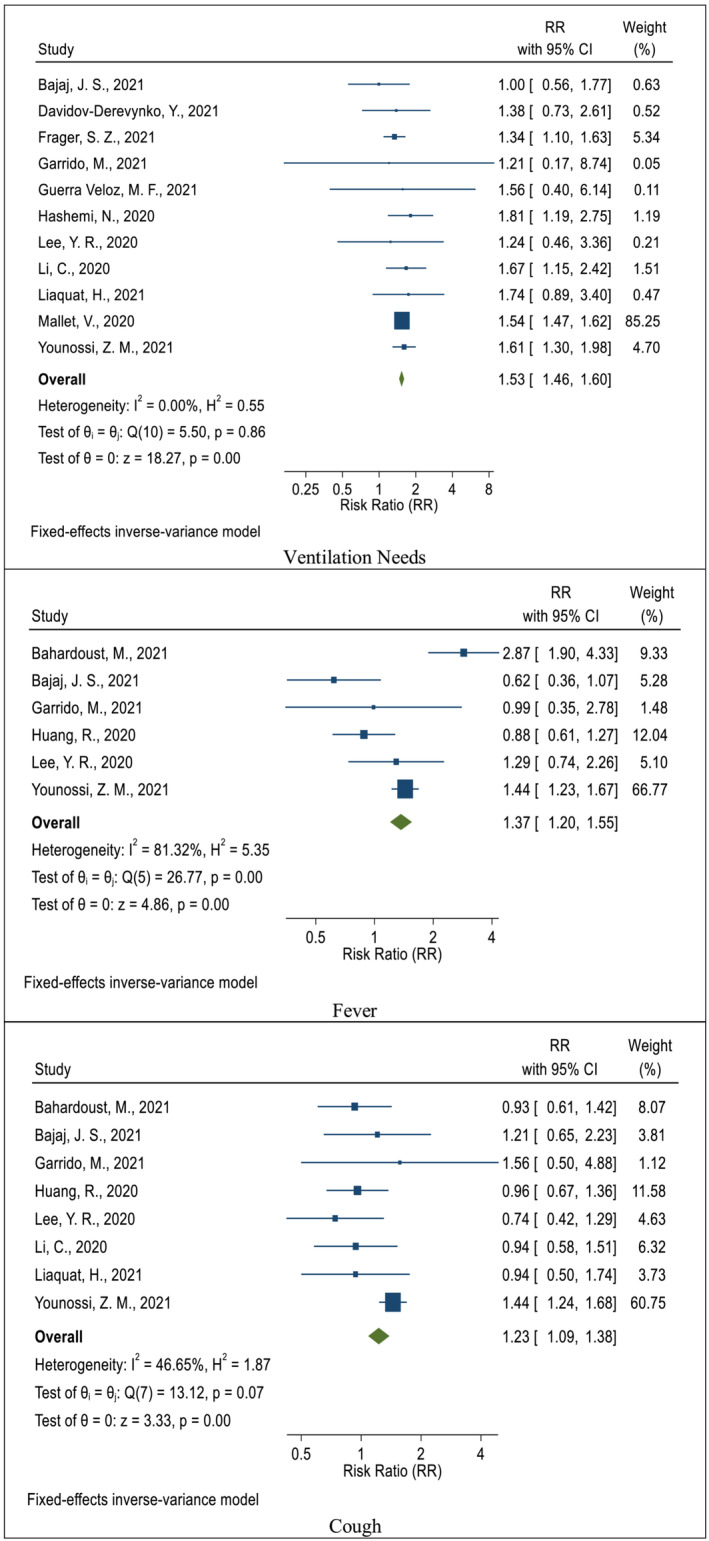
The pooled effect (risk ratio, RR) of chronic liver disorders on the ventilation needs, fever, and cough related to COVID‐19

The risk of need for ventilation in COVID‐19 patients who had the CLD was evaluated in 11 studies compared with those who did not have chronic liver disorders.^26^, ^29^, ^30^, ^32^, ^33^, ^34^, ^37^, ^38^, ^39^, ^40^, ^42^ In all of these studies, the need for ventilation was higher in the group with the CLD^26^, ^29^, ^30^, ^32^, ^33^, ^34^, ^37^, ^38^, ^39^, ^40^ The frequency of need for ventilation was higher in the group of patients with COVID‐19 and liver disorders.^42^ The cumulative RR after combining these studies was 1.53 (CI 95%: 1.46–1.60) with heterogeneity (*I* ^2^) equal to 0.2% (Figure 3).

To determine the association between the COVID‐19 symptoms and chronic liver disorders, patients with COVID‐19 who had chronic liver disorders were compared with those who did not have the CLD, and the results of the meta‐analysis showed cough, headache, myalgia, nausea, diarrhea and fatigue were 1.37 (CI 95%: 1.20–1.55), 1.23 (CI 95%: 1.09–1.38), 1.25 (CI 95%: 1.04–1.50), 1.19 (CI 95%: 1.02–1.40), 1.89 (CI 95%: 1.30–2.75), 1.49 (CI 95%: 1.07–2.09), and 1.14 (CI 95%: 0.98–1.33), respectively, while the risk of all these symptoms was higher in COVID‐19 patients with underlying liver diseases than ones without chronic liver disorders. Other symptoms have been reported in the table (Figure 3 and Table 3).

To examine the publication bias, the Eggers test was performed, which was not significant in all variables except myalgia and headache. That is, the publication bias has not occurred.

### 3.1 Subgroups analysis

The results of subgroups analysis according to age and continents for symptoms and complications related to COVID‐19 were reported in Table 4. The results show that complications related to COVID‐19 are more significant and severe in patients with CLD with age <60 and live in Asia (Table 4).

**TABLE 4:** The subgroups analysis of signs, symptoms, and complications related to COVID 19 in CLD patients based on continents and age

Outcomes	Outcomes	Subgroups	No. of study (SS)	Sample size liver disease	Sample size non‐liver disease	Pooled RR (95% CI)	Heterogeneity assessment		
							*I* ^2^	*P*‐value	Test Q
Outcomes of Covid‐19	Mortality	America	6(10068)	1400	8668	1.35 (1.19–1.53)	76.39%	0.00	21.18
		Asia	5(2632)	261	2371	2.66 (2.24–3.16)	84.49%	0.00	25.78
		Europe	4(260027)	15 579	244 488	1.49 (1.43–1.55)	63.62%	0.04	8.25
	ICU	America	4(3936)	693	3243	1.43 (1.25–1.65)	0.00%	0.71	1.38
		Asia	3(1668)	192	1475	1.34 (0.89–2.02)	0.00%	0.37	1.97
		Europe	2(640)	89	551	1.00 (0.61–1.64)	0.00%	0.37	0.82
	Ventilation	America	5(7288)	1150	6238	1.47 (1.29–1.67)	11.55%	0.34	4.52
		Asia	3(1491)	158	1333	1.55 (1.14–2.11)	0.00%	0.79	0.46
		Europe	3(259874)	15 518	244 356	1.54 (1.47–1.62)	0.00%	0.97	0.06
Covid‐19 symptoms	Fever	America	2(3434)	590	2844	1.35 (1.16–1.57)	88.05%	0.00	8.37
		Asia	3(2286)	214	2072	1.44 (1.13–1.84)	88.78%	0.00	17.83
		Europe	1(317)	14	303	0.99 (0.35–2.78)	‐	‐	‐
	Cough	America	3(3573)	624	2949	1.40 (1.21–1.61)	0.00%	0.37	1.98
		Asia	4(2390)	266	2124	0.91 (0.73–1.13)	0.00%	0.89	0.63
		Europe	1(317)	14	303	1.56 (0.50–4.88)	‐	‐	‐
Outcomes of Covid‐19	Mortality	<60	6(8007)	989	7018	1.89 (1.64–2.16)	75.76%	0.00	20.63
		>60	9(264760)	16 251	248 509	1.49 (1.44–1.55)	88.57%	0.00	69.99
	ICU	<60	5(5403)	773	4630	1.40 (1.21–1.62)	0.00%	0.72	2.08
		>60	4(840)	201	639	1.35 (1.05–1.75)	24.03%	0.27	3.95
	Ventilation	<60	5(5237)	739	4488	1.59 (1.34–1.89)	0.00%	0.97	0.49
		>60	6(263426)	16 087	247 339	1.53 (1.46–1.60)	0.00%	0.44	4.82
Covid‐19 symptoms	Fever	<60	3(4574)	686	3888	1.33 (1.16–1.53)	65.85%	0.05	5.86
		>60	3(1436)	132	1331	1.57 (1.15–2.15)	90.00%	0.00	20.00
	Cough	<60	4(4678)	738	3940	1.27 (1.12–1.45)	70.74%	0.02	10.25
		>60	4(1602)	166	1436	1.02 (0.76–1.37)	0.00%	0.78	1.09

### 3.2 Discussion

The RR of complications and consequences of COVID‐19 in patients with the CLD during the pandemic period was examined in this study and the results showed the need for ventilation and ARDS had higher risks than other outcomes related to COVID‐19 in patients with chronic liver disorders. Exacerbation of alveolar epithelial damages, increased vascular permeability, and systemic inflammation are among the factors that predispose to increased ARDS in patients with the CLD.^44^, ^45^ Also, because these patients had a higher BMI and obesity was more prevalent in them, their need for ventilation was higher when they were infected with COVID‐19.^46^

Due to the direct and significant effect of SARS‐CoV‐2 virus on ACE‐2 receptors in liver cells, patients with chronic liver disorders will have a more severe form of the disease if they develop COVID‐19, which causes their hospitalization in the intensive care unit of hospitals due to COVID‐19. According to the results of previous studies, patients with chronic liver disorders have a higher risk of being admitted to the intensive care unit than others in the community if they develop COVID‐19. In the present meta‐analysis, the same result was obtained, which was in line with the results of preliminary studies published in the world.^47^ However, the results of some studies in the world have not shown this association, for example, a study by Huang, R. et al. showed the risk of hospitalization in intensive care unit due to COVID‐19 in patients with chronic liver disorders was not significantly different from other people in the community.^35^ The reason for this inconsistency can be attributed to differences in the study method, how to collect information, and the type of patients studied in these articles.

Also, the risk of death due to COVID‐19 in the present meta‐analysis was significantly higher in patients with chronic liver disorders than other individuals. The reason for this can be attributed to the increase in inflammatory cytokines such as IL‐6, Ferritin and TNF‐alpha.^48^ In patients with COVID‐19, inflammatory cytokines increase, eventually leading to a cytokine storm in more severe forms of the disease. On the other hand, for patients with severe forms of COVID‐19, drugs that aggravate cytokines and cause cytokine storms are prescribed, which may also exacerbate death in COVID‐19 patients with chronic liver disorders. Coincidence of chronic liver disorders and the use of anti‐COVID‐19 drugs significantly increases the risk of mortality in COVID‐19 patients with the CLD. Also, worsening liver function reduces the number and disrupt the function of neutrophils, monocytes and innate immune proteins, and ultimately the number of both B and T lymphocytes involved in acquired immunity decreases and eventually immune dysfunction increases.^49^, ^50^

In this study, in addition to the mentioned cases, the symptoms associated with COVID‐19 including fever, cough, headache, myalgia, nausea, diarrhea, and fatigue were also evaluated in patients with the CLD. The results of this meta‐analysis showed among these symptoms, nausea, diarrhea and abdominal pain were more common than other ones in patients with chronic liver disorders. Increased gastrointestinal symptoms may be due to liver dysfunction. In addition, other symptoms such as fever, cough, headache, myalgia, and fatigue were more common in patients with the underlying liver disease than in healthy individuals while cytokines were effective in worsening these symptoms.^48^ The severity of various symptoms in patients with chronic liver disorders can affect outcomes of COVID‐19 and, as a result, increase the outcome and mortality in this group of patients.

Some studies on the effect of COVID‐19 on the incidence of liver disorders published results, which suggested in the case of COVID‐19 due to the widespread distribution of the main virus receptor called the angiotensin‐converting enzyme 2 (ACE2), the virus could cause a widespread disease with more involvement of extra‐pulmonary organs, especially the liver. ACE2 receptors are also expressed in the gastrointestinal tract, vascular endothelium, and hepatic cholangiocytes.^51^, ^52^ Various studies showed elevated liver enzymes indicated liver damages and were common in COVID‐19 patients with chronic and non‐CLDs.^52^ On the other hand, initial clinical studies in this area also confirmed a significant increase in liver enzymes such as ALT, and AST due to SARS‐CoV‐2 infection.^53^, ^54^, ^55^, ^56^ The results of a meta‐analysis also showed the levels of ALP, and γ‐GT enzymes were significant as a result of cholangiocellular damages.^57^, ^58^, ^59^ As the study results show, it is not yet clear how much an increase in liver enzyme levels can exacerbate the complications of COVID‐19 or the disease progression. In patients with COVID‐19 who have not had a chronic liver disorder or liver damage before infection, a slight liver disorder is found after recovery.^60^, ^61^

Abnormal results of liver tests were associated with more severe forms of COVID‐19 and mortality. Although RNAs of SARS‐CoV‐2 have been detected in the liver of patients with COVID‐19, it is not yet exactly clear how much SARS‐CoV‐2 infects the liver and multiplies in its cells.^17^, ^51^ The range of liver damages in the COVID‐19 disease may be direct infection by SARS‐CoV‐2, indirect involvement by systemic inflammation, and hypoxic changes. However, due to the major role of the liver in endobiotic and xenobiotic drug metabolism, coagulation, albumin, and production of acute phase reactants, liver dysfunction may affect the pathophysiology of the COVID‐19 disease.^62^, ^63^ The results of the present meta‐analysis along with other published results can be useful in finding many of these answers. They are also useful in updating treatment and prevention guidelines. This study was the first meta‐analysis to compare the complications and consequences associated with COVID‐19 in two groups of patients with the CLD and healthy ones. On the other hand, the results of subgroup analyses and overall results had a high homogeneity, which indicated the homogeneous and correct selection of initial studies in order to perform this meta‐analysis.

The heterogeneity in this meta‐analysis only was higher at two outcomes (Fever and Mortality). For detecting sources of this heterogeneity, all primary cohort studies were reviewed, and extracted related variables that reported in selected cohort studies completely. Between reported variables, age and continents were extracted. Other variables like type of underlying diseases, such as diabetes, coronary heart disease (CHD), and cancers, type of study population, type of measures tools for outcomes measure were not reported in selected cohort studies. The results of subgroup analysis based on age and continents show that the heterogeneity was decreased in many of categories, but in fever and mortality not decreased. This heterogeneity rate confirms the difference between the combined studies. This difference may be due to differences in some section of studies such as the methods of measuring the outcomes and the tools used, the methods of sampling, presence of important underlying diseases (comorbidities) like diabetes, CHD, cancers, and chronic obstructive pulmonary disease (COPD). These variables and factors not reported in selected cohort studies in this meta‐analysis, so authors could not subgroup analysis based on its.

The results of this research are also based on cohort studies, which are one of the most important observational studies in order to find a causal association, but the overall results of this study have not yet determined the exact association between SARS‐CoV‐2 infection and chronic liver disorders. In other words, according to the results of published studies, it can be claimed that there is a kind of causal association between these two factors, and in order to find it, studies based on genetics or molecular science such as Mendelian randomization are needed.

## 4 CONCLUSION

The results of this study showed the mortality and consequences due to COVID‐19 were significantly different between patients with the CLD and the general population. Also, the COVID‐19 symptoms in people with liver disorders were significantly more severe than those in healthy people. So, taking measures is necessary to manage them. It is recommended to reduce the risk of mortality and other consequences of COVID‐19 through screening and treating people with liver disorders in the lower stages of the CLD.

## CONFLICT OF INTEREST

The authors declare that they have no competing interests.

## AUTHOR CONTRIBUTIONS

YM: concept development (provided idea for the research). MA and PM: search strategy. MA, PM, and KZ: data extraction. YM: supervision. FM, SK, and MA: analysis/interpretation. All authors: writing (responsible for writing a substantive part of the manuscript).

## Data Availability

The datasets used and analyzed during the current study are available from the corresponding author on reasonable request.
